# FYN/TOPK/HSPB1 axis facilitates the proliferation and metastasis of gastric cancer

**DOI:** 10.1186/s13046-023-02652-x

**Published:** 2023-04-04

**Authors:** SanFei Peng, YuHan Yin, YiZheng Zhang, Feng Zhu, Ge Yang, Yang Fu

**Affiliations:** 1 Department of Gastrointestinal Surgery, The First Affiliated Hospital of Zhengzhou, University, Zhengzhou, 450052 China; 2 Cancer Research Institute, The Affiliated Hospital of Guilin Medical University, Guilin, 541000 Guangxi China; 3 Department of Ophthalmology, The First Affiliated Hospital of Zhengzhou University, Zhengzhou, 450052 China

**Keywords:** FYN, TOPK, Gastric Cancer Progression, Phosphorylation, HSPB1

## Abstract

**Background:**

FYN is a nonreceptor tyrosine kinase that regulates diverse pathological processes. The pro-cancer role of FYN in multiple malignancies has been elucidated. However, the mechanisms that FYN promotes gastric cancer (GC) progression remain largely unknown.

**Methods:**

In vitro and in vivo assays were used to investigate the function of FYN. FYN, TOPK, p-TOPK expression in GC specimens were detected by immunohistochemistry. Phosphoproteomics assays identify TOPK downstream substrate molecules. The molecular mechanism was determined using COIP assays, pull-down assays, immunofluorescence co-localization assays, western blotting, ^32^p-labeled isotope radioautography assays, vitro kinase assays, and TOPK knockout mice.

**Results:**

FYN was found to be significantly upregulated in GC tissues as well as in GC cells. Knockdown of FYN expression markedly attenuated the malignant phenotype of GC cells in vitro and in vivo. Mechanistically, we identified TOPK/PBK as a novel downstream substrate of FYN, FYN directly phosphorylates TOPK at Y272. One phosphospecific antibodies against Y272 was developed to validate the phosphorylation of TOPK by FYN. Moreover, the TOPK-272F mutation impaired the interaction between TOPK and FYN, leading to disappeared TOPK phosphorylation. Consistently, human GC tissues displayed increased p-TOPK(Y272), which correlated with poor survival. Phosphoproteomics results showed a significant downregulation of both HSPB1 and p-HSPB1(ser15) in TOPK-knockdown cells, which was confirmed by TOPK-konckout mice.

**Conclusions:**

FYN directly binds to TOPK in GC cells and phosphorylates TOPK at the Y272, which leads to proliferation and metastasis of GC. FYN-TOPK axis facilitates GC progression by phosphorylating HSPB1. Collectively, our study elucidates the pivotal role of the FYN-TOPK-HSPB1 cascade in GC.

**Supplementary Information:**

The online version contains supplementary material available at 10.1186/s13046-023-02652-x.

## Background

Gastric cancer is one of the most common malignant tumors worldwide, with the sixth highest incidence rate and the third highest mortality rate among all kinds of cancers [1]. With the rapid development of tumor biology, the molecular mechanism of GC development has been elucidated [2], and the advantages of targeted therapy are becoming apparent. Currently, the common molecular targets in GC are mainly protein kinases, including Her2 [3], VEGFR2 [4], etc. However, These targets are rarely effective in patients with GC due to molecular heterogeneity and acquired resistance [5]. Therefore, there is a need for new and effective targeted therapies.

FYN, also known as p59-FYN, Slk, Syn, is a 59 kDa protein containing 537 amino acids with genetic information located on chromosome 6q21 and it was originally identified as a member of the SFKs [6]. Dysregulation of FYN promotes tumorigenesis and progression of tumors through multiple biological functions, such as cell growth, apoptosis, migration, and drug resistance [7]. FYN has been shown to promote the progression of multiple cancers including GC [8–12]. However, the potential molecular mechanisms underlying the promotion of GC development by FYN are not known. Therefore, whether FYN can be an effective molecular target in GC needs to be further elucidated.

We found that inhibition of FYN expression inhibited the ability of GC cells to proliferate and metastasize. This is consistent with previous studies [12]. TOPK also has shown to be highly expressed in a variety of tumors such as ovarian cancer, breast cancer, gastric cancer, and colorectal cancer, and its expression correlates with tumor malignancy [13–17]. A large number of studies have confirmed that TOPK promotes cancer progression and therefore it is a promising potential therapeutic target [18–20]. We therefore tried to demonstrate the molecular mechanisms of FYN/TOPK in GC models.

In this study, we showed that endogenous TOPK and FYN can bind to each other in GC cells, and that FYN acts as an upstream regulatory molecule of TOPK to directly phosphorylate TOPK and thus regulate its molecular function. We also explored the downstream molecular network of TOPK through proteomics and phosphoproteomics, aiming to explain the mechanisms by which FYN/TOPK promotes GC progression. In summary, our data confirm that TOPK is a direct downstream substrate of FYN and that FYN promotes proliferation and metastasis of GC through phosphorylation of TOPK.

## Methods

### Antibodies and reagents

The β-actin (1:1000, 3700S) and p-Src Family (Tyr416) (E6G4R) (1:1000, #59548 s) antibodies were purchased from Cell Signaling Technology (Cell Signaling Technology, Beverly, MA, USA). HSPB1(T55934S), p-HSPB1(TA3080S) Flag antibody (1:5000, M20008s) and HA (1:5000, M20003s) were purchased from Abmart (Abmart Pharmaceutical Technology Co, Ltd.). FYN (1:1000, sc-73388, sc-434),TOPK(sc-293028) were purchased from Santa Cruz, Inc. (Santa Cruz Biotech.,USA).FYN(1:1000,ab184276,ab119855) were purchased from Abcam (Cambridge, UK). p-TOPK (Y272) antibody was prepared by Abiocode, Inc (shanghai, China). Rabbit (1:5000, GB21303) and mouse (1:5000, GB21301) antibodies were obtained from Servicebio, Inc. (Wuhan Servicebio Technology, Wuhan, China). We purchased OTS514 and HSP27 inhibitor J2 (Cat No. HY-18621, HY-124653) from MCE (MCE, China).

### Cell lines and culture condition

The human gastric cancer cell lines AGS, NCI-N87, KATO-III and Hs-746 T and HEK293T cell lines were purchased from American Type Culture Collection (ATCC; Manassas, VA, USA). The normal cell line GES-1 were purchased from China Center for Type Culture Collection (CCTCC), SGC7901, MGC803 cell line were purchased from Cell Bank of the Chinese Academy of Sciences (Shanghai, China). The cell lines were cultured in DMEM,1640 and F12K supplemented with 10% FBS at 37 °C, 5% CO2.

### Clinical data

The study materials consisted of 54 cases of gastric cancer were obtained from Department of Gastrointestinal Surgery, The First Affiliated Hospital of Zhengzhou University. This study was performed with the permission of the ethical committee of the First Affiliated Hospital of Zhengzhou University(2021-KY-0374–002).

### Preparation and purification of p-TOPK(Y272) antibody

The preparation of p-TOPK(Y272) antibody was done by Shanghai Abiocode Biotechnology Co. Phosphorylated and non-phosphorylated peptides required for antibody purification were synthesized by Gill Biochemical (Shanghai) Co., Ltd. Antibody purification is done with the Thermo Fisher SulfoLink™ Peptide Immobilization Kit (Item No. 44999), according to the kit protocol.

### Histopathology and Immunohistochemistry

Specimens were fixed in 10% formalin solution and embedded in paraffin wax. 4 μm serial sections were cut from the tissue blocks, deparaffinized in xylene, and hydrated in a series of alcohol (75%, 85%,95%, 100%), followed by antigen retrieval with EDTA. Tissue sections were then incubated with primary antibodies (FYN, TOPK and p-TOPK(Y272)). Subsequently, tissue sections were incubated with secondary antibody (Peroxidase-conjugated goat anti-rabbit Ig, ZB-2301, Zsbio, China; peroxidase-conjugated goat anti-mouse IgG, ZB-2305, Zsbio, China) for 2 h at room temperature, and then stained with DAB kit (ZL1 − 9018, ZSGB-BIO, China). After staining, sections were digitally scanned using the Aperio AT2 scanner (Leica Biosystems, Germany).

### Plasmids and shRNA

TOPK mutants at Y74, Y272, or Y74Y272 (designated Y74F, Y272F, and FF) were performed with the QuikChange Mutagenesis Kit (Stratagene, Inc., La Jolla, CA, USA). The mutant plasmids were sent to Sangon Biotech, Inc. (Shanghai, China) for DNA sequencing. The plasmids pcDNA3-HA-TOPK and pcDNA3 were provided by our laboratory. pcFLAG-FYN-WT was purchased from Addgene (USA). Two shRNA sequences were designed to knockdown FYN. These sequences are: 1. 5′-CCGGGTGCCAACAATCCTAGTGCTTCTCGAGAAGCACTAGGATTGTTGGCACTTTTT-3′;2.5′-CCGGCTGGAGAGACAGGTTACATTCCTCGAGGAATGTAACCTGTCTCTCCAGTTTTTTG-3′. Two shRNA sequences were designed to knockdown TOPK. These sequences are:1.5′-CCGGGAAGTGTGGCTTGCGTAAATACTCGAGTATTTACGCAAGCCACACTTCTTTTTG-3′;2.5′-CCGGGGGAACTAGGCCACCTATTAACTCGAGTTAATAGGTGGCCTAGTTCCCTTTTTG -3′.

### Bacterial expression and purification of the His-TOPK

PET-His-TOPK-WT and pET-His-TOPK-mutants were expressed in E. coli BL21 bacteria. Bacteria were grown at 37 °C to an absorbance of 0.6–0.8 at 600 nm, induced with 1 mM isopropyl β-D-thiogalactopyranoside (IPTG) at 30 °C for 4 h. All proteins were purified using nickel-nitrilotriacetic acid agarose (Qiagen, Inc., Valencia, CA, USA) overnight at 4 °C and eluted with 200 mM imidazole.

### In vitro kinase assay

The FYN active kinase and 10 × kinase buffer were purchased from Millipore Corp. (Billerica, MA, USA). The inactive substrate (2 μg) and the active kinase (0.2 μg in a 30 μl reaction) were incubated at 37 °C for 40 min in 1 × kinase buffer containing 100 μmol/L unlabeled ATP or 1 μCi [γ-32P] ATP. The samples were added with 5 × SDS buffer and then resolved by SDS-PAGE and visualized by autoradiography or Western blot.

### Western blot and coimmunoprecipitation

Cells (2 × 106) were seeded onto 10-cm-diameter dishes to 70–80% confluence and harvested in 200 μl RIPA buffer. The samples were separated on a 10% SDS-PAGE and subsequently transferred onto a PVDF membrane (Millipore, Billerica, MA, USA). Then antibody-bound proteins were detected by chemiluminescence (BIO-RAD, USA). Coimmunoprecipitation (CoIP) was performed using a CoIP Kit (Cat No.26147, Thermo Fisher Scientific, USA) according to the manufacturers instructions.

### Confocal laser scanning fluorescence microscopy

MGC cells were fixed in methanol (− 20 °C) and blocked in 5% normal goat serum at room temperature for 1 h. Then the cells were incubated overnight with the primary antibodies to detect TOPK and FYN at 4 °C. On the second day, the cells were incubated for 1 h at room temperature with the Alexa Fluor 546 (red for FYN) or Alexa Fluor 488 (green for TOPK) conjugated secondary antibody while being protected from light. Colocalization of proteins was observed by laser scanning confocal microscopy (NIKON C1si Confocal Spectral Imaging System, NIKON Instruments Co., Japan).

### Cell viability assay

CCK-8: Cell viability was determined using a cell counting kit-8 (CCK-8, Beyotime). Cells (5 · 103) were seeded in 96-well plates in Medium (100 ul) containing 10% FBS, and cultured overnight. After 24, 48, 72, and 96 h, CCK-8 solution (10ul) was added to each of the 96-well plates, and cultures were incubated for 90 min at 37 °C. Absorbance at 450 nm was measured using an automatic microplate reader (BioTeke, Beijing, China). MTT: Digestion, centrifugation and counting of cells to be assayed with a growth density of 80–90%, cells are grown in 96-well plates, Starting from the third day after plate laying, MTT assay was performed at the same time point every day; before the assay, MTT reagent(Sigma) and serum-free medium were prepared in the ratio of 1:9, the medium of the cells to be assayed was discarded, 100ul of the prepared mixture was added to each well and incubated in the cell incubator for 4 h, protected from light, After the incubation, 150ul of DMSO reagent was added to each well, shaken well and then the absorbance value of the cells at OD490 was detected using an enzyme marker, and the growth curve was calculated and plotted.

### Anchorage-independent cell transformation assay

Cells were seeded at 8 × 103 cells/per well into 6-well plates and cultured in 1 ml of 0.33% BME agar containing 10% FBS, with an additional 3 ml of 0.5% BME agar containing 10% FBS below. After the cells were cultured in a 37 °C, 5% CO2 incubator for 5–10 days, at which time colonies were observed by microscopy.

### Wound scratch assay

The cells were seeded in six-well plates in Medium and supplemented with 10% FBS. After the cells formed a confluent monolayer, a scratch was created in the center of the monolayer using a sterile p200 pipette tip. Next, the medium was removed and the cells were washed with PBS. The cells were subsequently incubated on a serum-free medium. The ability of cells to close the wound was assessed by comparing the 0,24,48 h phase-contrast micrographs of six marked points along the wounded area.

### Cell migration and invasion analysis

Migration and invasion abilities of transfected cells were evaluated via Transwell assay (8.0 µm pore size, 3422; Corning Incorporated, Corning, NY, USA). In brief, the lower chamber was coated by 600 μL Medium with 20% FBS, while 200 μL serum-free DMEM was added to the upper chamber. For invasion detection, Matrigel (356,234, BD Bioscience, USA) at a concentration of 2 mg/mL was added to the upper chamber. Transfected cells were seeded to the upper chamber at a concentration of 5 × 10^4^ cells/mL, and then the chamber was incubated for 24 h at 37℃ with 5% CO2. Later, the bottom chamber was fixed by 4% polyoxymethylene and stained with 0.01% Crystal Violet (Servicebio Technology Co., Ltd., Wuhan, China). Finally, the number of stained cells were counted under a microscope (DP74; Olympus Corporation, Tokyo, Japan). Six random views were selected for each sample.

### In vivo study

Female BALB/c nude mice (4–6 weeks of age) were used for all experiments. BALB/c nude mice were purchased from Beijing Vital River Laboratory Animal Technology, Beijing, China. In addition, All nude mice were maintained in SPF conditions at the Department of Zhengzhou University Animal Center strictly according to the institution’s guidelines. All animal work in this study was approved by the Ethics Committee of the Zhengzhou University Health Science Center (2,021,051,102).

For subcutaneous tumor formation experiments, 1 × 10^7^/200μL SGC7901 shmock or SGC7901 shFYN cells were digested and resuspended in sterilized PBS and then injected subcutaneously into BALB/c nude mice with 6 mice contained in each group. Tumor size was measured according to the formula: TV (mm3) = length × width^2^ × 0.5,after which tumors were excised and weighed. Finally, the tumors were harvested, photographed, and weighted. Sections were subjected to HE staining.

### Proteomic and phosphoproteomic analysis

shmock and shTOPK cell samples were sonicated three times on ice using a high intensity ultrasonic processor (Scientz) in lysis buffer (8 M urea, 1% protease inhibitor cocktail). Peptides were separated with a gradient of 2% to 60% acetonitrile in 10 mM ammonium bicarbonate pH 10 over 80 min into 80 fractions. The resulting MS/MS data were processed using MaxQuant search engine (v.1.6.15.0). Tandem mass spectra were searched against the human SwissProt database (20,422 entries) concatenated with reverse decoy database.It was performed with the support of Jingjie PTM Biolabs (Hangzhou,China) Co.Ltd.

### TOPK KnockOut(KO) mice

WT and TOPK KO mice were presented by Professor Zhu Feng from the Cancer Research Institute, Affiliated Hospital of Guilin Medical University. Design CRISPR targets based on the genome sequence structure of TOPK. The sheared DNA activity of the targets was detected by in vitro digestion of spCas91.1/gRNA. Targets with high activity will be selected for subsequent construction of gene mutant mice. Highly active gRNA targets were selected for in vitro transcription into RNA and microinjected into mouse fertilized eggs together with spCas91.1 protein to obtain first-build mice (founder) with mutations. By sequencing near the location of the CRISPR target, it was determined that the first mice carried the mutation and had fragment deletions. The first mouse with the mutation (founder) was mated with a wild-type mouse to obtain the F1 generation of mutant heterozygous mice ( ±), and DNA sequencing was performed to confirm that the target gene was fragment deleted and thus the target protein was inactivated, resulting in a knockout. Genotypically identical heterozygous mice ( ±) were mated with each other to obtain knockout pure heterozygous mice (-/-) F2 generation.

### Statistical analysis

All quantitative experiments were performed in triplicate at minimum. Statistical analysis was performed using Graphpad prism8.0. Student’s t-test was used to evaluate the data. Times for OS were defined from treatment initiation to date of death or last follow-up. The correlation between the level of FYN, TOPK or p-TOPK(Y272) and OS were assessed by log-rank test. In all tests, differences were considered significant at *P* < 0.05.

### Data and materials availability

The mass spectrometry proteomics data have been deposited to the ProteomeXchange Consortium via the PRIDE partner repository with the dataset identifier PXD036814.The other datasets used and/or analyzed during the current study are available from the corresponding author on reasonable request.

## Results

### FYN is highly expressed in GC patients and is closely associated with poor prognosis in GC patients

**Fig. 1:**
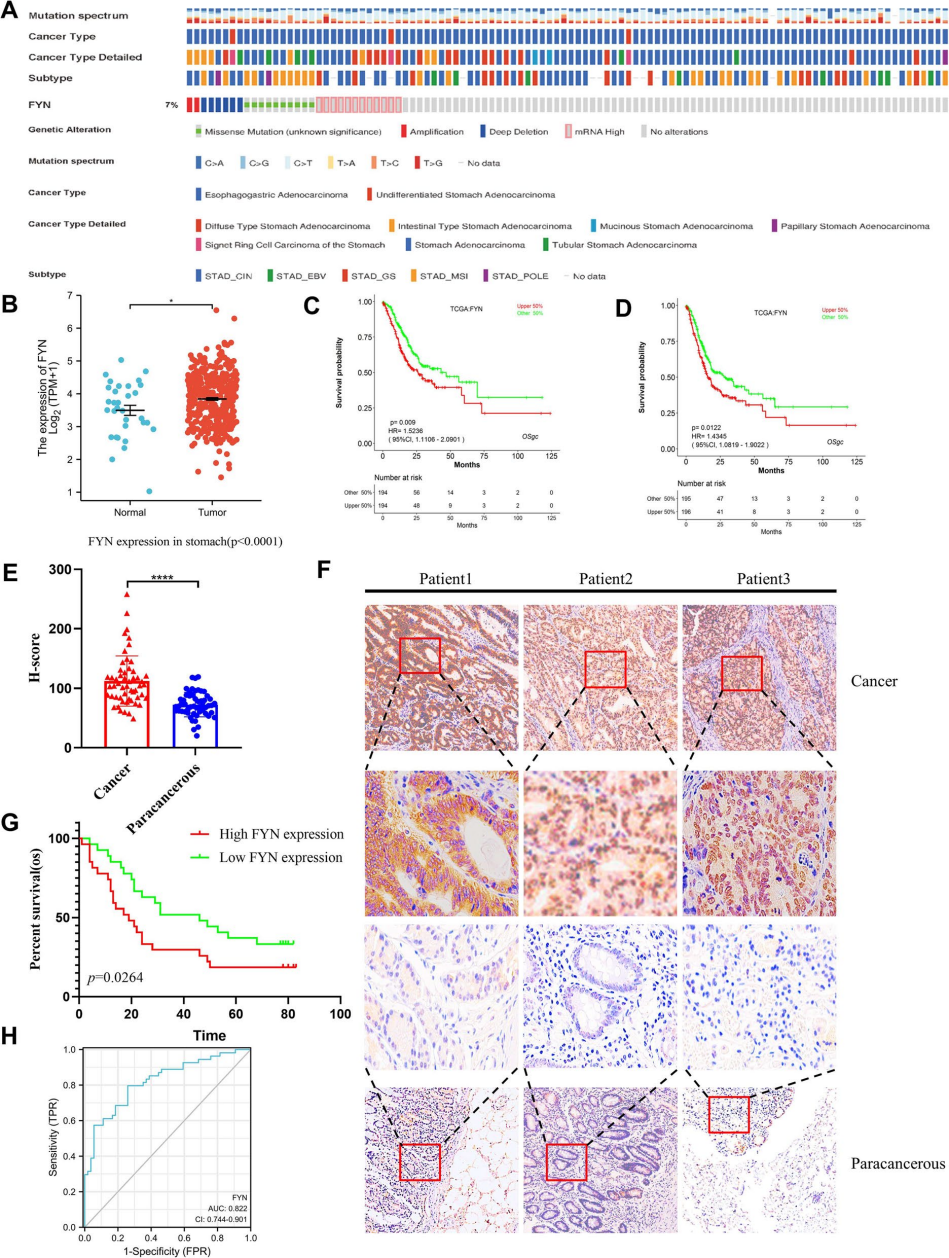
FYN is highly expressed in GC and is strongly associated with poor prognosis in GC patients. **A** The FYN genetic alterations (gene amplification, deep deletion, or somatic mutation) and mRNA expression in GC samples from the TCGA cohort (total alteration rate: 7%). **B** FYN mRNA expression is upregulated in the tumor tissues compared with it in normal tissue group from TCGA. **C** FYN mRNA high expression GC has poor overall survival. **D** FYN mRNA high expression GC has poor progression-free survival. **E**–**F** FYN expression was higher in gastric cancer tissues than in paraneoplastic tissues (*p* < 0.05). **G** Overexpressed FYN was associated with poor overall survival in GCs (*p* < 0.05).**H**. Specificity of ROC curve to detect FYN as a diagnostic marker for GC

First we detected the expression of FYN in Cbioportal. We found that the amplification and copy number gain of FYN is a common event in GC cases from The Cancer Genome Atlas (TCGA) cohort (Fig. 1A). Additionally, FYN mRNA level was upregulated in GC tumor samples compared with it in normal tissues from TCGA (Fig. 1B). Then we analyzed the expression of FYN in different stages—T stage, N stage, M stage, pathologic stage, age, and gender respectively. We found that the mRNA levels of FYN were higher in T stage (T3&T4), N stage (N2&N3), M stage (M1), pathologic stage (III&IV), and higher in females than in males, without any significant correlation with age (Fig. S1A-F). Next, to further explain the role of FYN in the progression of GC, we performed GSEA enrichment analysis using TCGA GC dataset grouped by FYN expression level and found that the molecules in the high FYN expression group were mainly enriched in several major proto-oncogenic signaling pathways (Fig. S1G-H). After that we analyzed the effect of FYN mRNA expression on the prognosis of GC patients, and found that patients with high FYN expression GC had worse overall survival (OS) and progression-free survival (PFS) (Fig. 1C, D). Finally, we analyzed the protein levels of FYN in GC and normal tissues in the human protein altas database and found that FYN protein levels were higher in GC tissues than in normal tissues (Fig. S1I). Therefore, we speculate that FYN may play a pro-cancer role in GC. To further verify the expression of FYN in GC and its impact on the prognosis in GC patients, we collected immunohistochemical analysis of cancer and paracancerous tissue specimens from 54 GC patients. The clinicopathological characteristics of the patients are shown in Table 1, FYN expression was found to be significantly higher in cancerous tissues compared with paracancerous tissues, with statistically significant differences (Fig. 1E, F). Following IHC analysis, we then carried out survival analysis and found that the expression of FYN was closely related to the prognosis in GC patients, and the overall survival of GC patients in the group with high expression of FYN was significantly shorter. The median survival of patients with high expression FYN GC was 19 months, and it was 46 months in patients with low expression FYN (Fig. 1G). ROC curves were used to evaluate the FYN expression test as gastric cancer diagnosis and to demonstrate sensitivity and specificity (AUC = 0.822 (95% CI = 0.744–0.901), Fig. 1H).

**Table 1:** Patient characteristics and associations with FYN expression in GC patients

Characteristic	FYN high expression	FYN low expression	p
n	27	27	
status, n (%)			0.352
0	5 (9.3%)	9 (16.7%)	
1	22 (40.7%)	18 (33.3%)	
gender, n (%)			0.271
female	14 (25.9%)	9 (16.7%)	
male	13 (24.1%)	18 (33.3%)	
T stage, n (%)			0.342
T1b	2 (3.7%)	1 (1.9%)	
T2	3 (5.6%)	1 (1.9%)	
T3	15 (27.8%)	16 (29.6%)	
T4a	5 (9.3%)	9 (16.7%)	
T4b	2 (3.7%)	0 (0%)	
N stage, n (%)			0.337
N0	5 (9.3%)	9 (16.7%)	
N1	2 (3.7%)	3 (5.6%)	
N2	6 (11.1%)	6 (11.1%)	
N3a	11 (20.4%)	9 (16.7%)	
N3b	3 (5.6%)	0 (0%)	
M stage, n (%)			1.000
M0	26 (48.1%)	26 (48.1%)	
M1	1 (1.9%)	1 (1.9%)	
Clinical Stage, n (%)			0.815
1A	1 (1.9%)	1 (1.9%)	
1B	1 (1.9%)	1 (1.9%)	
2A	4 (7.4%)	7 (13%)	
2B	2 (3.7%)	3 (5.6%)	
3A	5 (9.3%)	1 (1.9%)	
3B	9 (16.7%)	9 (16.7%)	
3C	4 (7.4%)	4 (7.4%)	
4	1 (1.9%)	1 (1.9%)	
Pathologic stage, n (%)			1.000
II	7 (13%)	7 (13%)	
II-III	4 (7.4%)	4 (7.4%)	
III	16 (29.6%)	16 (29.6%)	
H-score(FYN), meidan (IQR)	108.5 (100.81, 125.34)	62.25 (53.14, 72.79)	< 0.001
time, meidan (IQR)	19 (11.5, 47.5)	46 (20.5, 77)	0.052
age, mean ± SD	56.93 ± 14.52	59.81 ± 8.88	0.383
Number of lymph node—positive, meidan (IQR)	8 (2, 13)	3 (0, 7)	0.038

### Knockdown of FYN significantly inhibited the proliferation and metastatic capacity of gastric cancer cells

**Fig. 2:**
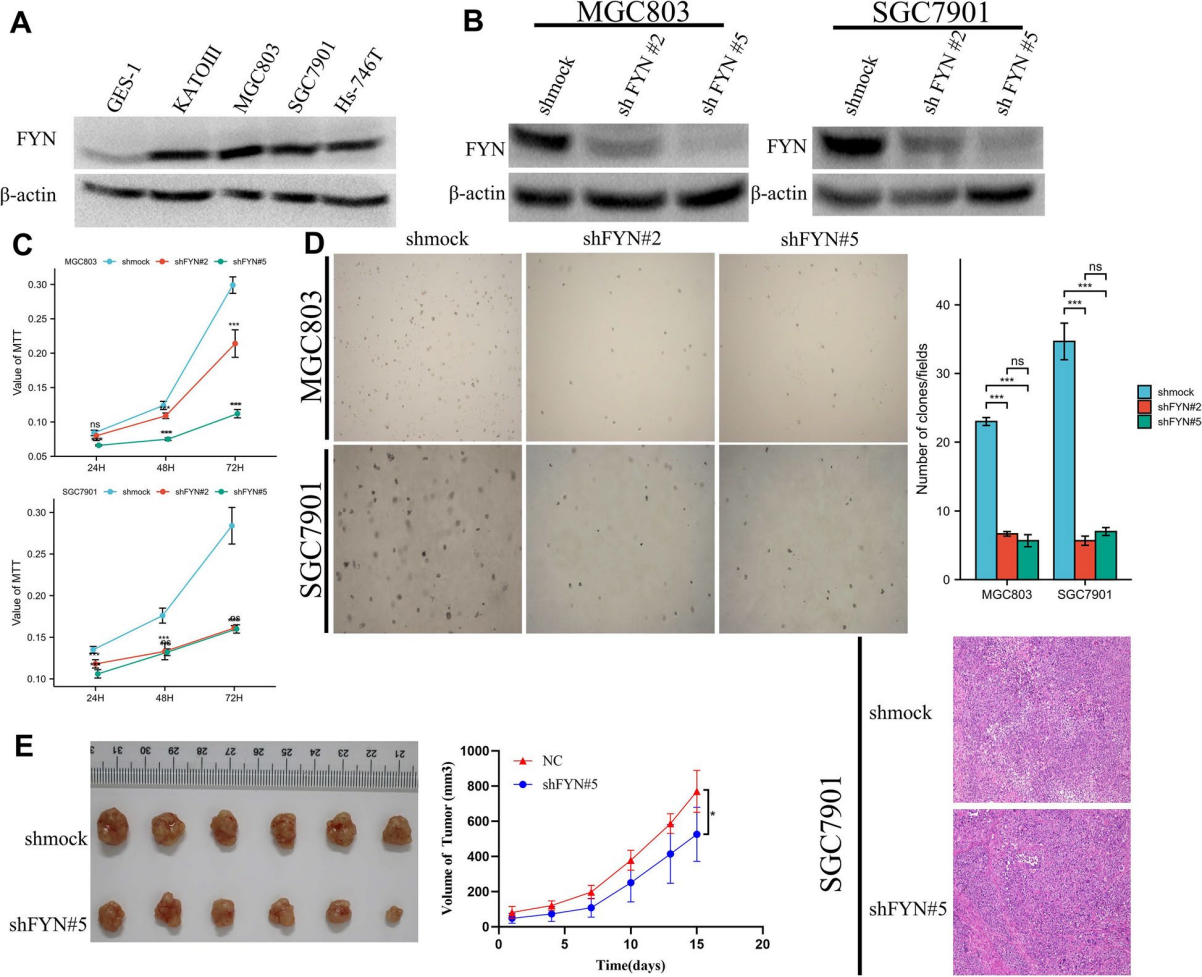
Knockdown of FYN inhibited the proliferation of gastric cancer cells. **A** Expression of FYN in four strains of gastric cancer cells and normal stomach epithelial cells. **B** Knockdown of FYN expression in SGC7901 and MGC803 cells. **C** MTT assay confirmed that inhibition of FYN expression significantly inhibited the proliferation of SGC7901 and MGC803 cells. 5 × 10^3^ cells per well, *N* = 3, The data are expressed as the means ± standard deviations. *p < 0.05, **p < 0.01, ***p < 0.001, ns not significant. **D** Softagar assay confirmed that knockdown of FYN significantly inhibited the clone formation ability of MGC803 and SGC7901 cells. 8 × 10^3^ cells per well, *N* = 3, The data are expressed as the means ± standard deviations. *p < 0.05, **p < 0.01, ***p < 0.001, ns not significant. **E** In vivo experiments confirmed that inhibition of FYN significantly inhibited the growth rate of tumors. 1 × 10.^7^ cells per mouse, *N* = 6. Tumor size was measured according to the formula: TV (mm3) = length × width2 × 0.5. *N* = 6. The data are expressed as the means ± standard deviations. *p < 0.05, ns not significant (Student’s T-test)

**Fig. 3:**
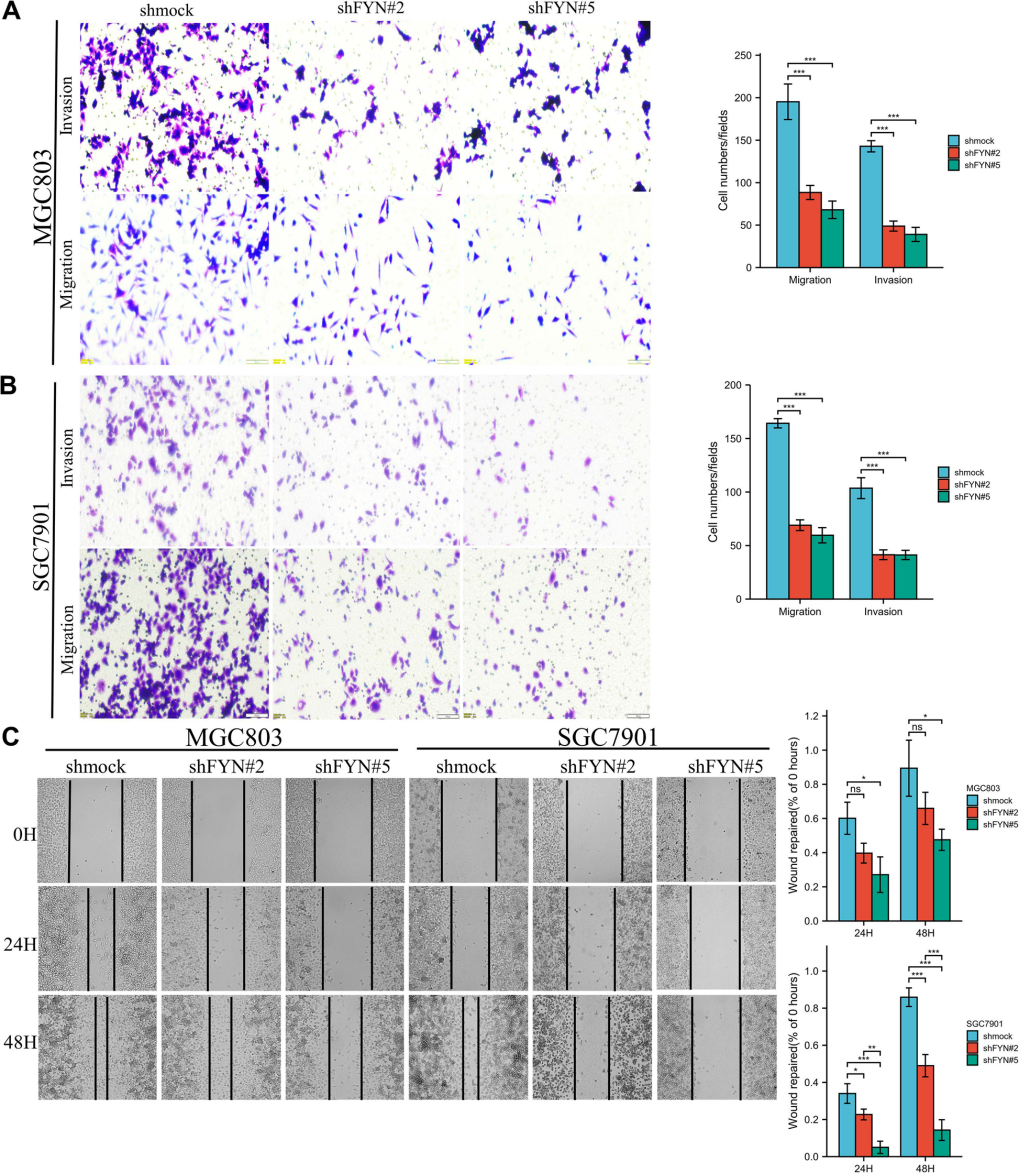
Knockdown of FYN inhibits the migration and invasion of gastric cancer cells. **A** Knockdown of FYN inhibited the migration and invasion ability of MGC803 cells. The number of invaded cells per well was quantified. scale bar, 100 μm. *N* = 5, The data are expressed as the means ± standard deviations. *p < 0.05, **p < 0.01, ***p < 0.001, ns not significant. **B** Knockdown of FYN inhibited the migration and invasion ability of SGC7901 cells. The number of invaded cells per well was quantified. scale bar, 100 μm. *N* = 5, The data are expressed as the means ± standard deviations. *p < 0.05, **p < 0.01, ***p < 0.001, ns not significant. **C** Scratch assay confirmed that knockdown of FYN significantly inhibited the migration ability of MGC803 and SGC7901 cells. The scratch area of 0H, 24H, 48H is calculated and quantified. *N* = 3, The data are expressed as the means ± standard deviations. *p < 0.05, **p < 0.01, ***p < 0.001, ns not significant

We next examined the protein expression levels of FYN in gastric normal cells GES-1 and four GC cell lines. The results showed that FYN was expressed at higher levels in SGC7901 and MGC803 cells than in GES-1 cells (Fig. 2A). Therefore, we knocked down the expression of FYN in SGC7901 and MGC803 cells (Fig. 2B). The proliferation of gastric cancer cells was significantly inhibited when FYN was reduced. (Fig. 2.C, D). In vivo experiments also confirmed that silencing FYN inhibited tumor growth, and H&E results showed that cells in the control group proliferated more actively than those in the shFYN group (Fig. 2E). Additionally, the reduction in FYN expression also significantly inhibited gastric cancer cell migration and invasion (Fig. 3A-C).

### TOPK is the bona fide substrate of FYN, FYN directly binds TOPK and phosphorylates TOPK at Y272

**Fig. 4:**
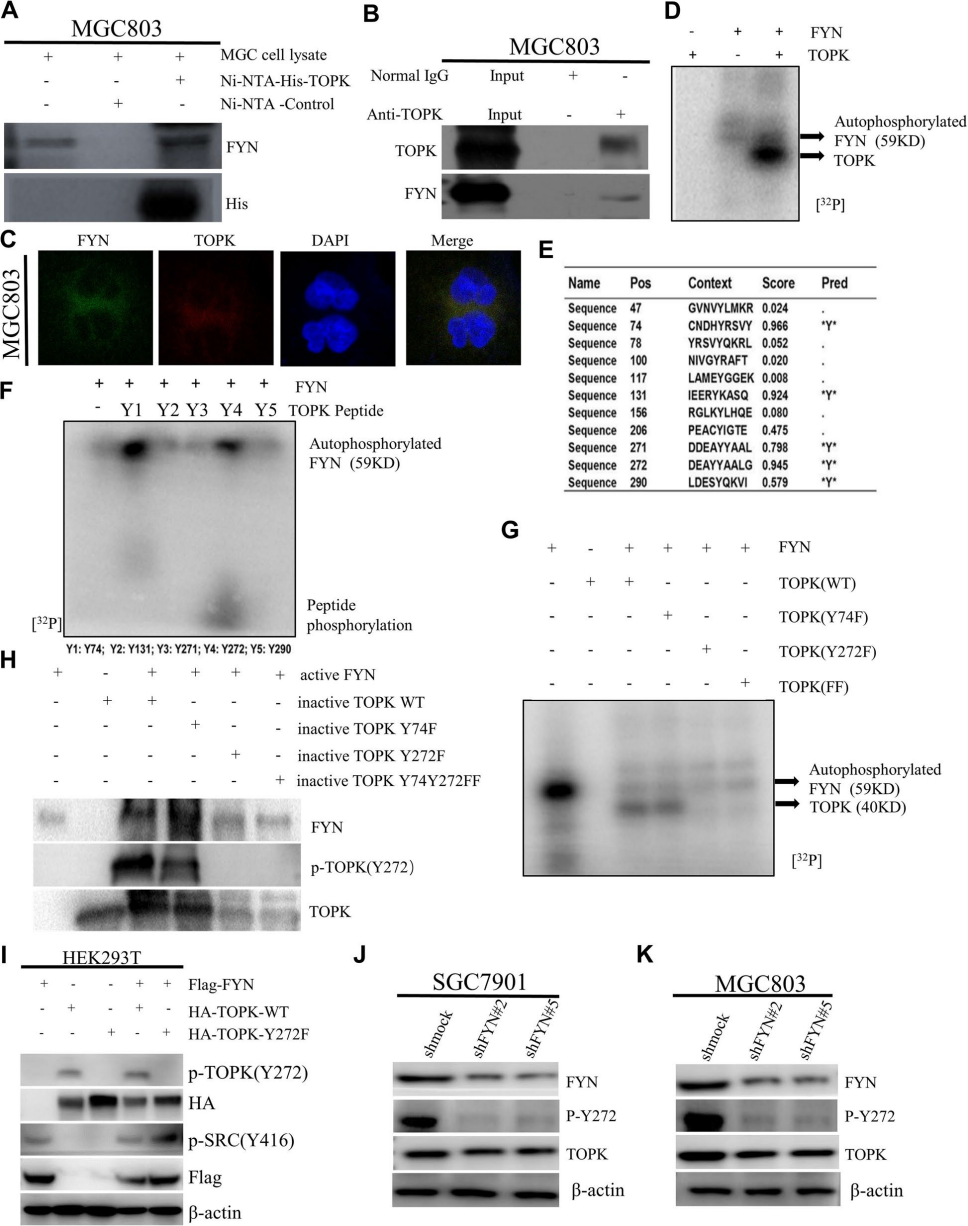
FYN directly binds TOPK and phosphorylates TOPK at Y272. **A** Ni–NTA-His-TOPK bound with endogenous FYN of MGC803 cells. **B** COIP experiments confirm that FYN can bind to TOPK endogenously. **C** Colocalization of FYN and TOPK was visualized by confocal microscope in MGC803 cells. Cytoplasmic and nuclear staining of FYN and TOPK was mostly merged together. **D** Active FYN phosphorylated inactive TOPK in vitro in the presence of [γ-32P] ATP as visualized by autoradiograph. **E** Potential phosphorylated tyrosine sites of TOPK were predicted by NetPhos 3.1 software program. **F** FYN phosphorylated TOPK at Y272 in peptide mapping. Five synthesized peptides containing potential tyrosine sites were used as substrates in an in vitro kinase assay with active FYN in the presence of [γ-32P] ATP and the results were visualized by autoradiography. **G** Wild type His-TOPK (WT), single mutant His-TOPK (74F), single mutant His-TOPK (272F) or double mutant His-TOPK (FF) were used as substrates in an in vitro kinase assay with active FYN in the presence of [γ-32P] ATP and the results were visualized by autoradiography. **H** Validation of anti phospho-TOPK (Y272) (p-TOPK (Y272)) in an in vitro kinase assay. Wild type His-TOPK (WT), single mutant His-TOPK (74F), single mutant His-TOPK (272F) or double mutant His-TOPK (FF) as shown was used as substrate for active FYN. Reactive products were resolved by SDS-PAGE and visualized by Western blot with p-TOPK (Y272). **I** Co-transfection of Flag-FYN and HA-TOPK-WT,HA-TOPK-Y272F in 293 T cells. **J**-**K** Detection of changes in TOPK, p-TOPK(Y272) levels after knockdown of FYN expression in SGC7901 and MGC803 cells

After confirming that FYN can promote the proliferation and migration ability of GC cells, we next searched for the molecular mechanism by which FYN affects the progression of GC. Since the authors of this study were under the guidance of Prof. Zhu F who first explained the molecular mechanism of TOPK in cancer in 2007 [16], subsequent studies have been centered on the need to activate TOPK. Prof. Zhu F and colleagues also demonstrated that SRC of the same family of FYN could directly bind and phosphorylate TOPK to promote colon carcinogenesis [13]. Therefore, we speculated whether FYN could directly bind and phosphorylate TOPK. We performed pull-down assay using His-TOPK-Ni–NTA-Agarose constructed successfully in Prof. Zhu F's lab in FYN high expression cell line and found that FYN could bind TOPK (Fig. 4A). Then we performed immunoprecipitation assay with anti-TOPK antibody (COIP) which revealed direct binding between FYN and TOPK in MGC803 cells (Fig. 4B). Finally, we performed immunofluorescence co-localization in MGC803 cells with green fluorescence for FYN and red fluorescence for TOPK and revealed that FYN could co-localize with TOPK spatially (Fig. 4C) and bind directly to TOPK. We next performed ^32^p-labeled isotope radioautography experiments using active FYN and inactivated TOPK proteins which showed that TOPK can be phosphorylated by active FYN (Fig. 4D). To find the sites where TOPK could be phosphorylated by FYN, we used Netphos 3.1 to predict the five most likely tyrosine sites Y74, Y131, Y271, Y272, Y290 (Fig. 4E), and then we synthesized the corresponding peptides and performed ^32^p-labeled isotope radioautography experiments with active FYN and the five peptides, which showed that the Y272 could be phosphorylated (Fig. 4F). Next we mutated the inactivated TOPK-WT plasmid to TOPK-Y74F, TOPK-Y272F, TOPK-Y74Y272F and again performed ^32^p-labeled isotope radioautography experiments and found that the phosphorylation signal of TOPK-Y272F, TOPK-Y74Y272F disappeared. However, TOPK-WT and TOPK-Y74 phosphorylation signals did not disappear (Fig. 4G). To confirm this result, we made p-TOPK(Y272) antibody for another in vitro kinase assay, and the results were consistent with the previous result (Fig. 4H). FYN was cotransfected with TOPK-WT and TOPK-Y272F in 293 T cells, and the results showed that the p-TOPK(Y272) signal was enhanced after cotransfection of FYN and TOPK-WT, while the p-TOPK(Y272) signal was lost after cotransfection of FYN and TOPK-Y272F (Fig. 4I). We examined the effect of FYN silencing on p-TOPK(Y272) signaling in SGC7901 and MGC803 cells with FYN silencing, and the results showed that p-TOPK(Y272) decreased after FYN silencing (Fig. 4J, K). The above results indicated that FYN could directly bind and phosphorylate TOPK at the Y272. The enhanced proliferation and invasion ability of GC cells caused by FYN may be mediated by TOPK, and a new phosphorylation site of TOPK was identified.

### Mutation of the TOPK tyrosine 272 or the use of TOPK inhibitors can eliminate the ability of FYN to produce pro-migrated and invasive GC cells.

**Fig. 5:**
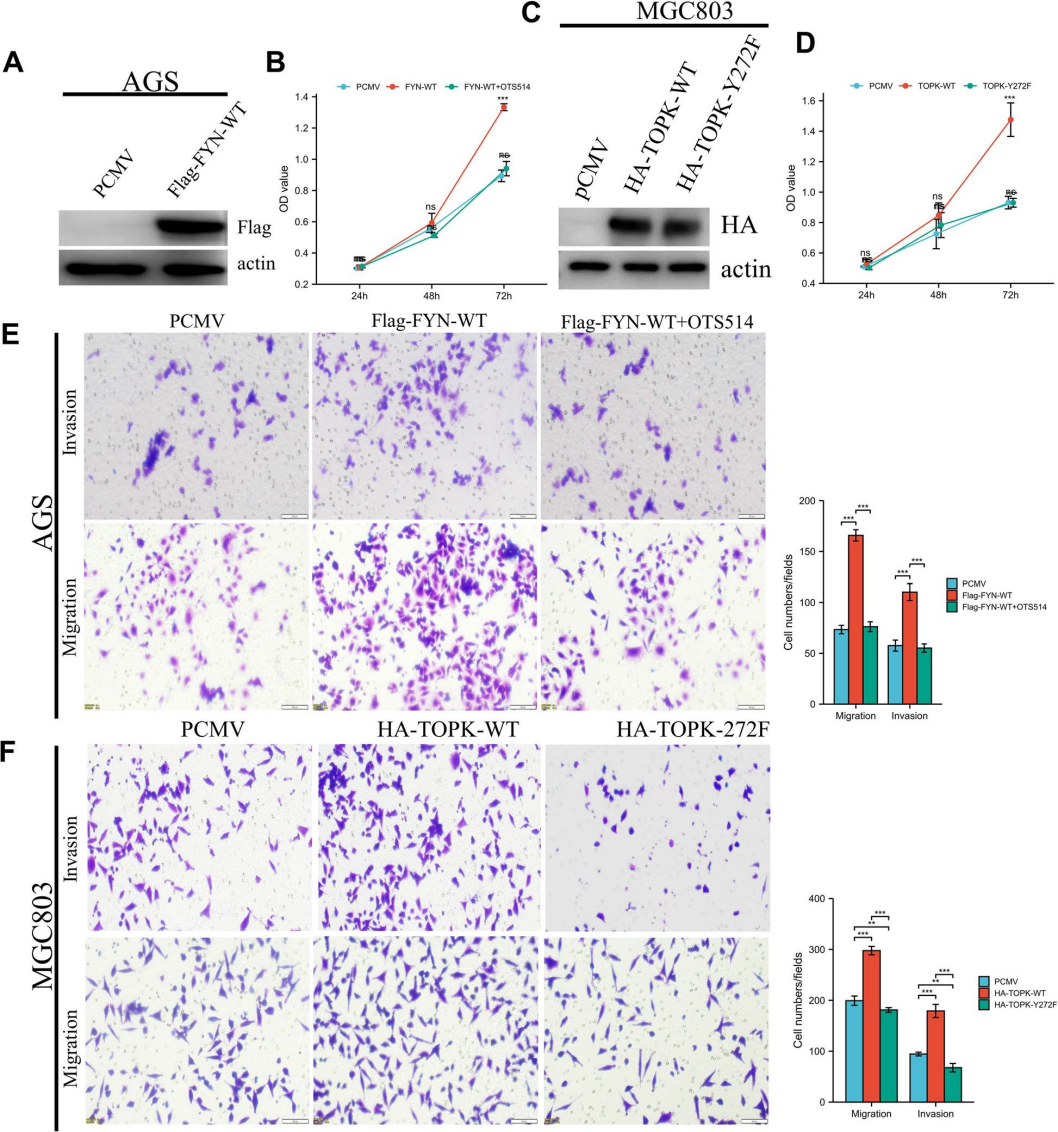
TOPK mediates FYN to promote gastric cancer proliferation and metastasis. **A** Overexpression of FYN in AGS cells. **B** CCK-8 assay for OD value of PCMV, OE-FYN and OE-FYN + OTS514 groups. 5 × 10^3^ cells per well, *N* = 4, The data are expressed as the means ± standard deviations. *p < 0.05, **p < 0.01, ***p < 0.001, ns not significant. **C** Overexpression of TOPK-WT and TOPK-Y272F in MGC803 cells. **D** CCK-8 assay for OD value of PCMV, TOPK-WT and TOPK-Y272F groups. 5 × 10^3^ cells per well, *N* = 4, The data are expressed as the means ± standard deviations. *p < 0.05, **p < 0.01, ***p < 0.001, ns not significant. **E** Transwell assay to determine the difference between PCMV, OE-FYN and OE-FYN + OTS514 groups. The number of invaded cells per well was quantified. scale bar, 100 μm. *N* = 5, The data are expressed as the means ± standard deviations. *p < 0.05, **p < 0.01, ***p < 0.001, ns not significant. **F** Transwell assay to determine the difference between PCMV, TOPK-WT and TOPK-Y272F groups. The number of invaded cells per well was quantified. scale bar, 100 μm. *N* = 5, The data are expressed as the means ± standard deviations. *p < 0.05, **p < 0.01, ***p < 0.001, ns not significant

After confirming that FYN phosphorylates TOPK at the Y272 by in vitro kinase assays and in vivo experiments, we next tested whether the enhanced proliferation or metastatic capacity of GC cells induced by FYN was mediated by TOPK by rescue experiment. We first established FYN overexpression cell lines in the FYN low expression cell line AGS (Fig. 5A), and then we performed CCK-8 assays with PCMV, OE-FYN, and OE-FYN + OTS514. The results showed that the OE-FYN group was more proliferative than the PCMV group, while the addition of TOPK inhibitor OTS514 in the OE-FYN group inhibited the proliferative ability induced by FYN. The above results indicate that the enhanced proliferation ability of GC cells induced by FYN was mediated by TOPK, and the addition of TOPK inhibitor could reverse the pro-proliferative ability of FYN cells (Fig. 5B). Next, we used migration and invasion assays to verify whether the pro-metastatic ability of FYN-induced GC cells was mediated by TOPK, and the results showed that the metastatic ability of cells in the OE-FYN group was significantly enhanced compared with the PCMV group, while the addition of TOPK inhibitor OTS514 reversed this phenomenon (Fig. 5E). The above results suggest that the ability of FYN to promote the proliferation and metastasis of GC cells is generated by phosphorylating TOPK. To confirm the importance of the Y272 on the biological function of TOPK, we established TOPK-WT and TOPK-Y272F overexpression cell lines in TOPK low expressing MGC803 cells (Fig. 5C). Next, we performed CCK-8 and transwell assay. The CCK-8 results showed that the TOPK-WT group had significantly enhanced proliferation ability compared with the control group, while the TOPK-Y272F group showed no significant difference compared with the control group (Fig. 5D). Transwell results showed that the number of cells entering the lower chamber in the TOPK-WT group was significantly higher than that in the PCMV group, while the number of cells entering the lower chamber in the TOPK-Y272F group was not significantly different from that in the PCMV group (Fig. 5F). The above results indicate that TOPK can enhance the proliferation and metastasis of GC cells in gastric cancer and is a pro-cancer molecule, while the Y272 is critical for TOPK to exercise its biological function.

### Phosphoproteomics to identify TOPK downstream substrates

**Fig. 6:**
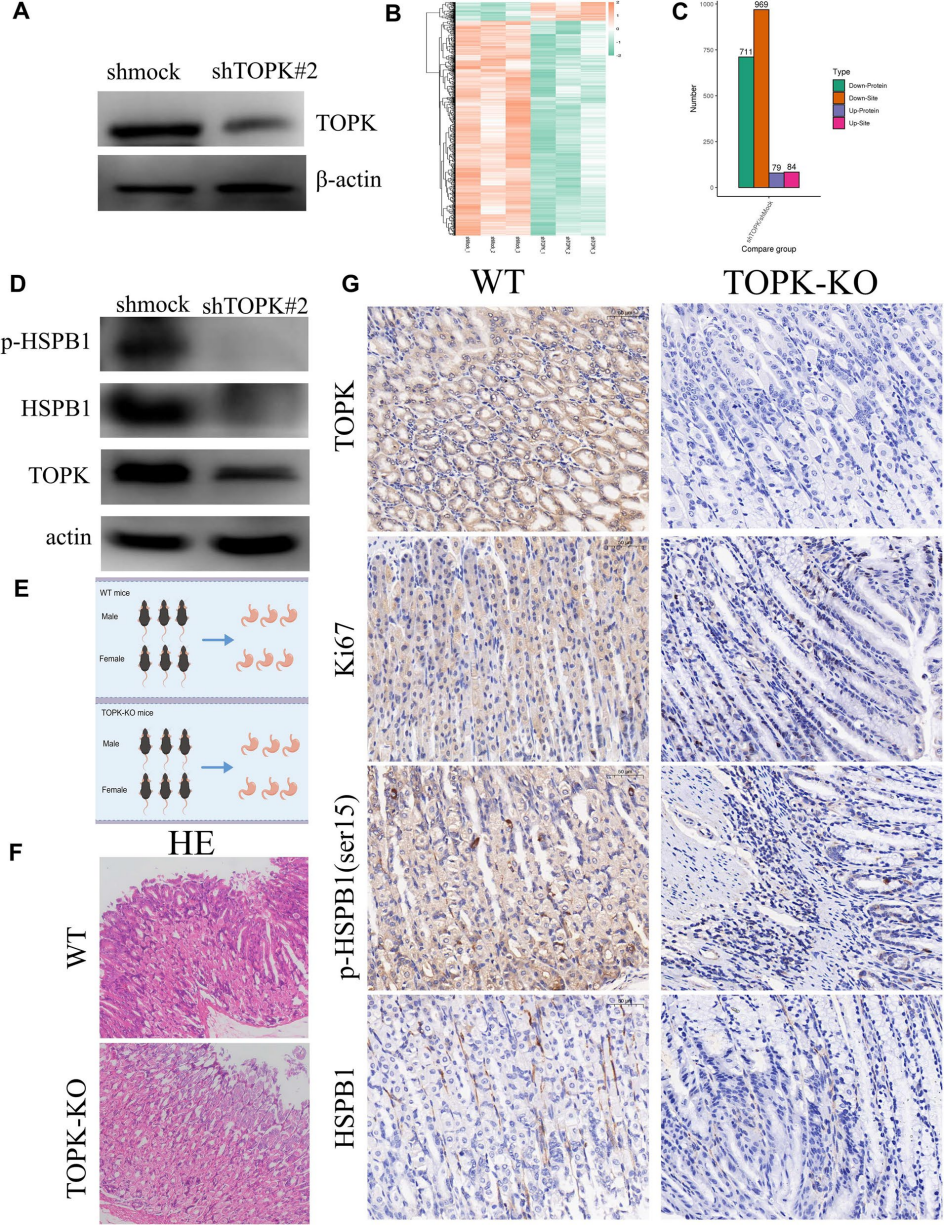
Validation of downstream substrate molecules screened by phosphoproteomics. **A** Knockdown of TOPK expression in AGS cells. **B** Heatmap of differentially phosphorylated proteins. **C** Summary of differentially phosphorylated proteins and loci. **D** Western blot validation of the differences in HSPB1 protein and phosphorylation levels in AGS shmock and shTOPK cells. **E** Diagram of TOPK-KO mice (By Figdraw). **F** HE staining of gastric tissues of WT and TOPK KO mice. **G** IHC of gastric tissues from WT and TOPK KO mice to verify the expression levels of TOPK, Ki67, HSPB1 and p-HSPB1 (ser15)

After confirming TOPK as a downstream substrate of FYN, we next identified the downstream molecular network regulated by TOPK. We first silenced TOPK in AGS cells with high TOPK expression (Fig. 6A) and then we examined the molecular signaling network altered by silencing TOPK through proteomics combined with phosphoproteomics (Fig. S2A). With the heat map results showing the downstream protein molecules and phosphorylation sites altered by knocking down TOPK (Fig. 6B). We found that the number of proteins with down-regulated phosphorylation levels after TOPK silencing was 711 and the number of down-regulated sites was 969, and the number of proteins with up-regulated phosphorylation levels was 79 and the number of up-regulated sites was 84 (Fig. 6C). Then we selected the proteins with down-regulated phosphorylation levels for COG enrichment analysis and GO terms enrichment analysis and found that they were mainly enriched in signaling mechanism, the cytoskeleton, protein post-translational modifications, protein turnover and cell cycle control and other biological processes (Fig. S2B, C). Also, we evaluated the characteristic motifs of phosphorylation peptides using the Motif-X algorithm. According to the scores derived from Motif-X, xxxxxD_S_ExExxx, xxxxxx_S_xDEExx, xxxxxx_S_DEDxxx, xxxxxx_S_DDDxxx were the top four ranked characteristic motifs (Fig. S2D). Next we identify the phosphorylation motifs of TOPK-regulated phosphorylated proteins and plotted a heat map of 20 amino acids near the Serine modification residues. We also identify the phosphorylation motifs of TOPK-regulated phosphorylated proteins, we plotted a heat map of 20 amino acids near the Threonine modification residues. Also, we evaluated the characteristic motifs of phosphorylation peptides using the Motif-X algorithm. According to the scores derived from Motif-X, xxxxxx_T_DxExEx, xxxRxx_T_PPxxxx, xxxxxx_T_DExxxx, xxxxxx_T_PPxxxx were the top four ranked characteristic motifs (Fig. S2E). They are new and unique. These phosphoproteins were subdivided into 4 groups according to experimental/control group ratio values and named Q1 to Q4, and then enriched for biological processes, cellular components, molecular functions and protein structural domains (Fig. S3A-C).The down-regulated proteins were mainly in Spliceosome signaling pathway, HIPPO signaling pathway and VEGF signaling pathway (Fig. S3D-F). Indicating the main altered KEGG signaling pathway after TOPK downregulation was the inhibition of pro-cancer signaling pathway and the activation of cancer suppressor signaling pathway. Finally, we selected HSPB1 with significantly and markedly decreased protein levels and phosphorylation levels for validation in vitro and in vivo. The phosphoproteomics results showed that HSPB1 could be phosphorylated by TOPK at Ser15, HSPB1 antibody and p-HSPB1(ser15) antibody were used to verify in TOPK knockdown cell lines. Both HSPB1 and p-HSPB1(ser15) appeared to be significantly decreased in the AGS shTOPK group compared with the AGS shmock group (Fig. 6D). To make the results more convincing, we constructed TOPK knockout (KO) mice, and took gastric tissues of six mice each from WT group and TOPK-KO group for HE staining, compared with TOPK-KO group, WT group showed more active cell proliferation (Fig. 6E,F). Next, we performed immunohistochemistry for TOPK, Ki67, HSPB1 and p-HSPB1(ser15) in WT and TOPK-KO groups, and the results showed that Ki67, HSPB1 and p-HSPB1(ser15) were not significantly expressed in gastric tissues of TOPK-KO group compared with WT group (Fig. 6G). The results were consistent with phosphoproteomics data.

### HSPB1 acts as a downstream effector of FYN/TOPK and mediates its biological function

**Fig. 7:**
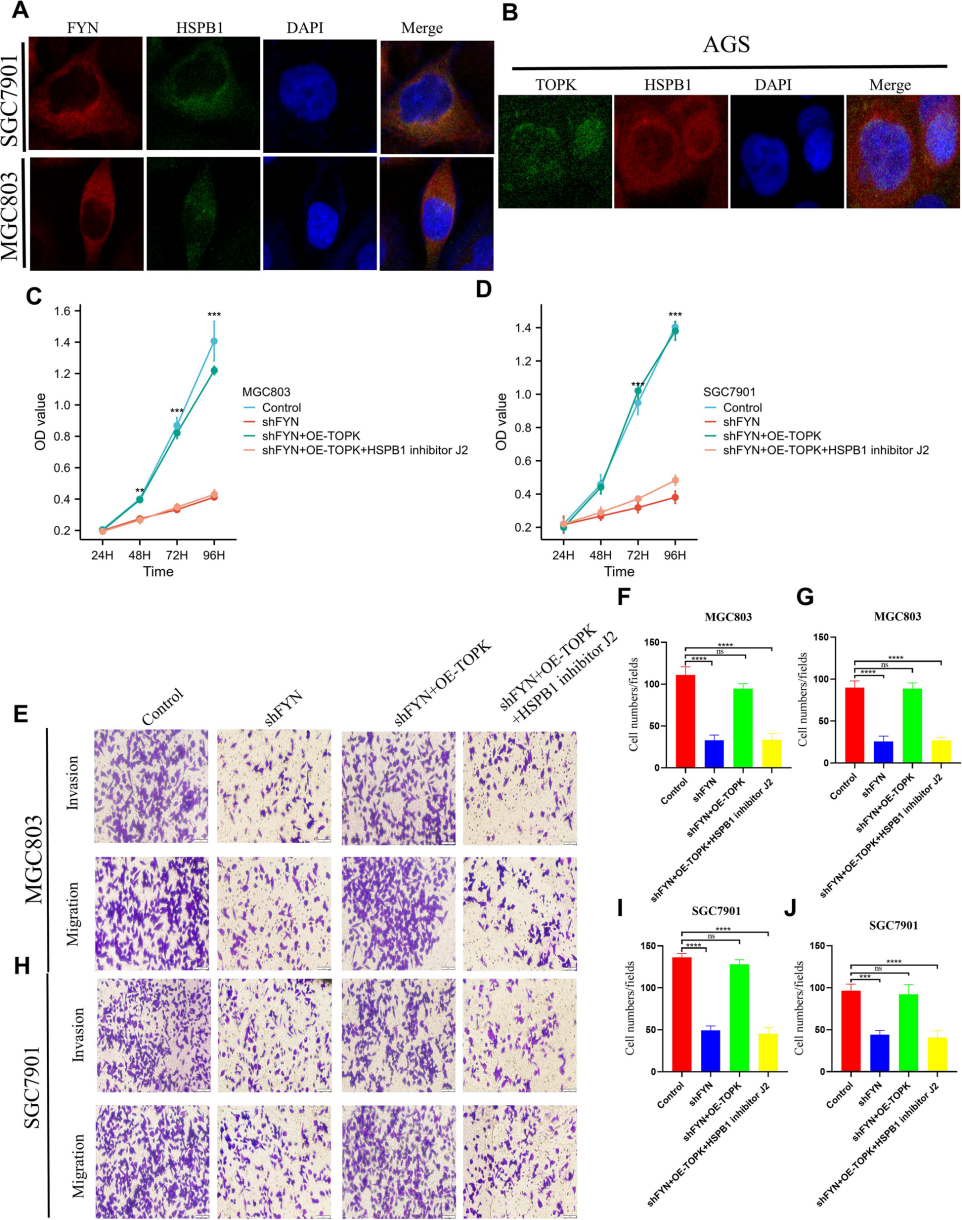
HSPB1 acts as a downstream effector of FYN/TOPK and mediates its biological function. **A** Colocalization of FYN and HSPB1 was visualized by confocal microscope in MGC803 and SGC7901 cells. Cytoplasmic and nuclear staining of FYN and TOPK was mostly merged together. **B** Colocalization of TOPK and HSPB1 was visualized by confocal microscope in AGS cells. **C**-**D** CCK-8 assay detected cell viability in Control group, shFYN group, shFYN + OE-TOPK group and shFYN + OE-TOPK + HSPB1 inhibitor J2 group. 5 × 10^3^ cells per well, *N* = 3, The data are expressed as the means ± standard deviations. *p < 0.05, **p < 0.01, ***p < 0.001, ns not significant. **E** Representative images of migration and invasion of Control group, shFYN group, shFYN + OE-TOPK group and shFYN + OE-TOPK + HSPB1 inhibitor J2 group in MGC803 cells. **F** Statistical results of the number of migrated cells in each group of MGC803 cells. The number of invaded cells per well was quantified. scale bar, 100 μm. *N* = 5, The data are expressed as the means ± standard deviations. *p < 0.05, **p < 0.01, ***p < 0.001, ****p < 0.0001, ns not significant. **G** Statistical results of the number of invasive cells in each group of MGC803 cells. The number of invaded cells per well was quantified. scale bar, 100 μm. *N* = 5, The data are expressed as the means ± standard deviations. *p < 0.05, **p < 0.01, ***p < 0.001, ****p < 0.0001,ns not significant. **H** Representative images of migration and invasion of Control group, shFYN group, shFYN + OE-TOPK group and shFYN + OE-TOPK + HSPB1 inhibitor J2 group in SGC7901 cells. **I** Statistical results of the number of migrated cells in each group of SGC7901 cells. The number of invaded cells per well was quantified. scale bar, 100 μm. *N* = 5, The data are expressed as the means ± standard deviations. *p < 0.05, **p < 0.01, ***p < 0.001, ****p < 0.0001, ns not significant. J. Statistical results of the number of invasive cells in each group of SGC7901 cells. The number of invaded cells per well was quantified. scale bar, 100 μm. *N* = 5, The data are expressed as the means ± standard deviations. *p < 0.05, **p < 0.01, ***p < 0.001, ****p < 0.0001, ns not significant

To further clarify whether FYN or TOPK physically binds to HSPB1, we performed co-localization analysis of FYN and HSPB1 in MGC803 and SGC7901 cells, and examined the binding of TOPK and HSPB1 in AGS cells. The results identified the co-localization of FYN or TOPK with HSPB1 in the cytoplasm (Fig. 7.A, B). We next performed an analysis of whether HSPB1 acts as a FYN/TOPK downstream effector mediating FYN/TOPK to produce biological functions, and we performed rescue assays in the Control group, shFYN group, shFYN + OE-TOPK group and shFYN + OE-TOPK + HSPB1 inhibitor J2 group, respectively, by performing CCK -8 and Transwell experiments. The results showed that overexpression of TOPK reversed the decreased proliferation, migration and invasion ability caused by knockdown of FYN compared to the shFYN group, while addition of HSPB1 inhibitor J2 partially reversed this behavior (Fig. 7.C-J).

### Increased expression of TOPK and p-TOPK(Y272) in GC patients is associated with poor prognosis of GC patients

**Fig. 8:**
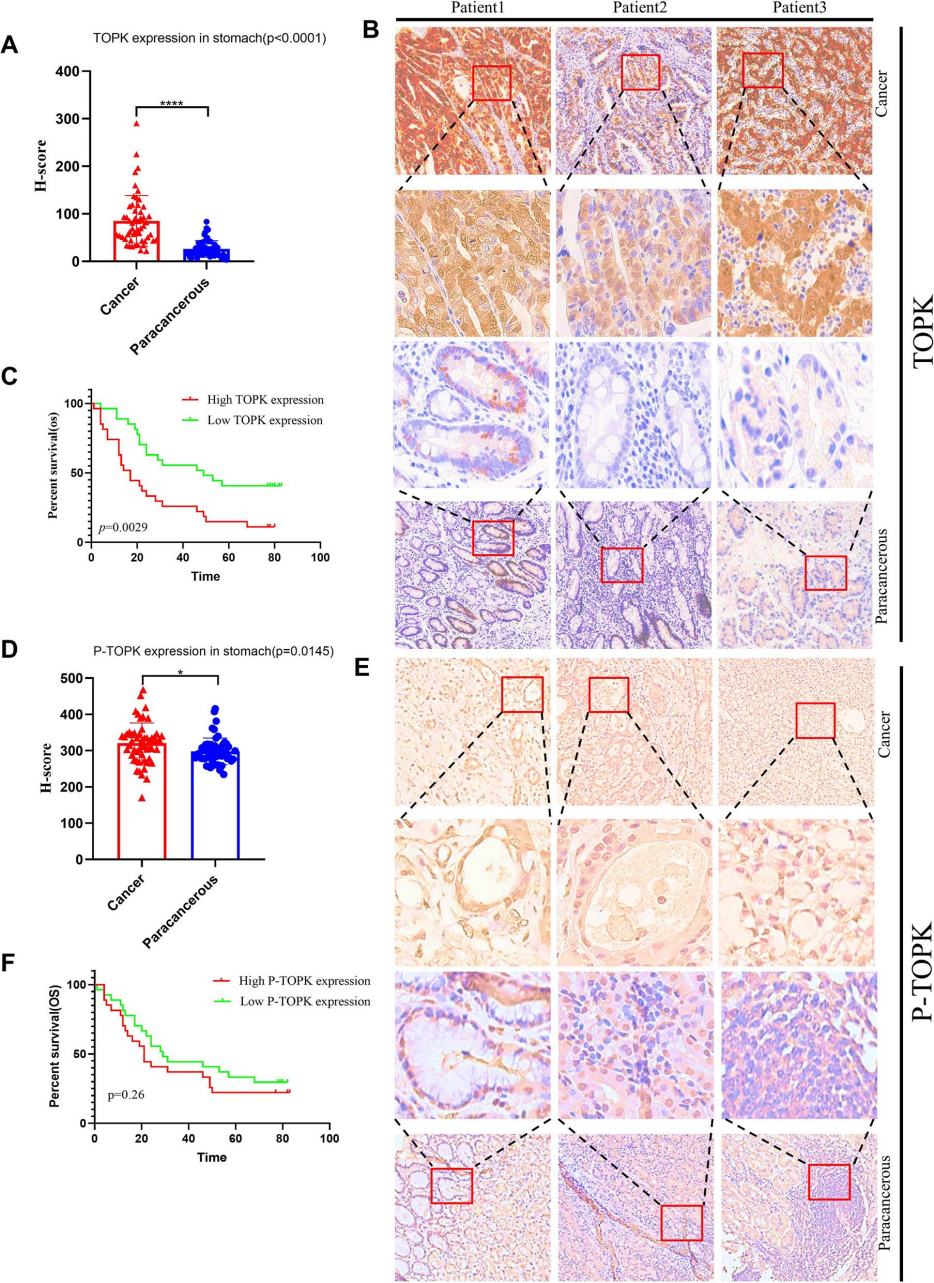
Overexpression of TOPK and p-TOPK(Y272) in gastric cancer patients is associated with poor prognosis of gastric cancer patients. **A**-**B** TOPK expression was higher in gastric cancer tissues than in paraneoplastic tissues (*p* < 0.05). **C** Overexpressed TOPK was associated with poor overall survival in GCs (*p* < 0.05). **D**-**E** p-TOPK(Y272) expression was higher in gastric cancer tissues than in paraneoplastic tissues (*p* < 0.05). **F** Overexpressed p-TOPK(Y272) was associated with poor overall survival in GCs

We found that the amplification and copy number gain of TOPK is a common event in GC cases from The Cancer Genome Atlas (TCGA) cohort (Fig. S4A). Additionally, TOPK mRNA level was upregulated in GC tumor samples compared with that in normal tissues from TCGA (Fig. S4B). Then we analyzed the expression of TOPK in different T stages, N stages, M stages, Pathologic stages, Age and Gender respectively (Fig. S4C-H). To verify the relationship between TOPK, p-TOPK expression in clinical samples and prognosis of GC patients, we performed IHC on the same cohort of GC patients (Table 1) and found that TOPK protein was significantly more highly expressed in GC tissues than in normal tissues, with statistically significant differences (Fig. 8A, B). We divided TOPK into high and low expression groups according to the median H-score value of TOPK, and analyzed the relationship between TOPK expression level and prognosis of GC patients by KM method, and found that the high TOPK expression group had a poor prognosis compared with the low expression group (Fig. 8C). p-TOPK expression was significantly higher in GC tissues compared with normal tissues (Fig. 8D, E). The relationship between the expression level of p-TOPK and the prognosis of GC patients was analyzed by the same method and it was found that the prognosis of GC patients with high expression of p-TOPK was worse than that of GC patients with low expression of p-TOPK (Fig. 8F). The results of clinical samples show that TOPK and p-TOPK are expressed at elevated levels in GC, patients with high expression of TOPK and p-TOPK have shorter survival, and TOPK as a downstream molecule of FYN is also a cancer-promoting factor in GC.

## Discussion

Src family kinases (SFKs) are overexpressed in various cancers and have long been proposed as molecular targets for therapy [21]. FYN is a member of the SRC family and plays a proto-oncogenic function in multiple tumors. It has been demonstrated that FYN promotes the proliferation and metastasis of oral cancer, colorectal cancer, breast cancer, lung cancer, pancreatic cancer, cholangiocarcinoma [10, 11, 22–27]. However, FYN is downregulated and acts as a tumor suppressor in prostate cancer [28]. In addition, it has been demonstrated that high expression of activated FYN induces differentiation and growth arrest in neuroblastoma cells, yet patients with neuroblastoma with high expression of FYN have long-term survival [29]. Thus, FYN may play a dual role in the development of cancer. In the current study, we retrieved TCGA-GC data for analysis and found that FYN mRNA was highly expressed in GC tissues and was closely associated with common cancer-promoting signaling pathways, and patients with high FYN expression had significantly shorter OS and PFS. Therefore, we hypothesized that FYN plays a pro-cancer function in GC, and clinical samples from our hospital verified this conclusion. Moreover, silencing of FYN in gastric cancer cells significantly inhibited cell proliferation, migration and invasion, and nude mice tumorigenic and metastatic models also confirmed that FYN could promote GC proliferation and metastasis. This provides strong evidence for FYN as a pro-cancer factor in GC.

T-LAK cell-originated protein kinase (TOPK), also known as PDZ-binding kinase (PBK), is a dual specificity serine/threonine kinase, plays a vital role in a variety of cellular functions [18]. Moreover, dysregulation of TOPK expression and activity in cancer leads to cancer progression [20]. The function of TOPK in cancer may be regulated by multiple sites and there are other upstream kinases to be discovered, which may be related to the heterogeneity of cancer. Cyclin B1/CDK1 phosphorylates TOPK at the Thr9 site and thereby activates substrates to promote cell division [30]. Prof. zhu F and colleagues also demonstrated that MET, as an upstream molecule of TOPK, directly phosphorylates TOPK at the Y74, resulting in reduced apoptosis in gefitinib-resistant NSCLC cells, which in turn is involved in the development of drug resistance [31]. Janus kinase 2 (JAK2) mediates Burkitt lymphoma growth by phosphorylating TOPK at Y74 [32]. TOPK is also a direct downstream substrate of ERK2, which is activated by direct phosphorylation of TOPK at S32, and after activation, TOPK plays an important role in sorafenib resistance in renal cell carcinoma (RCC) [33]. In our study, the application of TOPK inhibitor OTS514 in FYN overexpressing cells could significantly inhibit the proliferation and migration ability of GC cells induced by FYN. This suggests that TOPK may act as a downstream substrate of FYN. TOPK was also highly expressed in GC tissues and strongly correlated with poor prognosis. This is consistent with previous studies [17]. However, the precise mechanism regarding the interaction of FYN with TOPK is not clear. We confirmed that FYN and TOPK directly bind in cells and phosphorylate TOPK at Y272 to promote the proliferation and metastasis of GC cells. Phosphorylation by FYN at position Y272 promotes the proliferation and metastatic ability of GC cells. Y272 phosphorylation is essential for TOPK to promote GC progression. In our study, we confirmed the pro-cancer role of FYN/TOPK axis in GC progression, and inhibition of FYN or TOPK could significantly delay the progression of GC.

TOPK acts as an oncogenic factor mainly by activating downstream molecular substrates or proto-oncogenic signaling pathways. It has been demonstrated that phosphorylation of H3 at the S10 site by TOPK promotes proliferation of breast cancer [34], phosphorylation of PRPK at the S250 site by TOPK promotes metastasis of colon cancer [35], and phosphorylation of c-Jun at the S63/73 site promotes the development of drug resistance in lung cancer [36]. However, in addition to the above identified TOPK downstream substrates, in GC TOPK may promote cancer progression through several new unknown downstream substrates. Therefore, we mapped the downstream molecular network of TOPK by proteomics combined with phosphoproteomics, aiming to explain the molecular signaling pathways affected by TOPK in GC. We found that HSPB1 was significantly downregulated at both protein level and Ser15 phosphorylation level in TOPK-KD group. The results were confirmed with HSPB1 antibody and p-HSPB1 (Ser15) in TOPK-KO mice and TOPK-KD cells, suggesting that HSPB1 may be a downstream effector of TOPK. HSPB1 may be phosphorylated at Ser15 by TOPK to produce the malignant phenotype caused by FYN. Heat shock protein family B (small) member 1 (HSPB1, also known as HSP27), a member of the low molecular weight heat shock protein family. HSPB1 is also a molecular chaperone whose main function is the stabilization of abnormally folded proteins and the regulation of cytoskeletal organization [37, 38]. The upstream protein kinases that have been identified to phosphorylate HSPB1 were PKC, PKD and MAPKAPK-2/3 [39–41]. However, HSPB1 is phosphorylated to inhibit apoptosis [42] and is closely associated with ferroptosis [43]. According to recent studies, HSPB1 can form a p38/pMAPKAPK2/pHSPB1 cascade with p38/MAPKAPK2 in GC to encourage tumor growth and metastasis [44]. HSPB1 has also been demonstrated to play a vital role in a wide variety of cancer [45–48]. Using phosphoproteomics, we identified that the Ser15 of HSPB1 is phosphorylated by TOPK and confirmed this result in both cells and animals. The above results suggest that HSPB1 may act as a direct downstream substrate of TOPK to mediate the progression of GC induced by the FYN/TOPK axis. Meanwhile TOPK may be another new upstream kinase of HSPB1, which is unreported.

According to our findings, FYN accelerates GC progression by phosphorylating TOPK at Y272 to cause this pro-carcinogenic effect. When TOPK is inhibited or Y272 mutated, the procarcinogenic actions of FYN are reversed, suggesting that Y272 is essential for TOPK. It also confirmed for the first time that FYN and TOPK play a key role as partners in the development and metastasis of GC. In addition, HSPB1, a key effector molecule downstream of the FYN/TOPK axis, was also identified, and the FYN-TOPK-HSPB1 cascade was established in GC. Therefore, based on our current findings, targeted inhibition against FYN/TOPK/HSPB1 may benefit GC patients.

## Conclusions

**Fig. 9:**
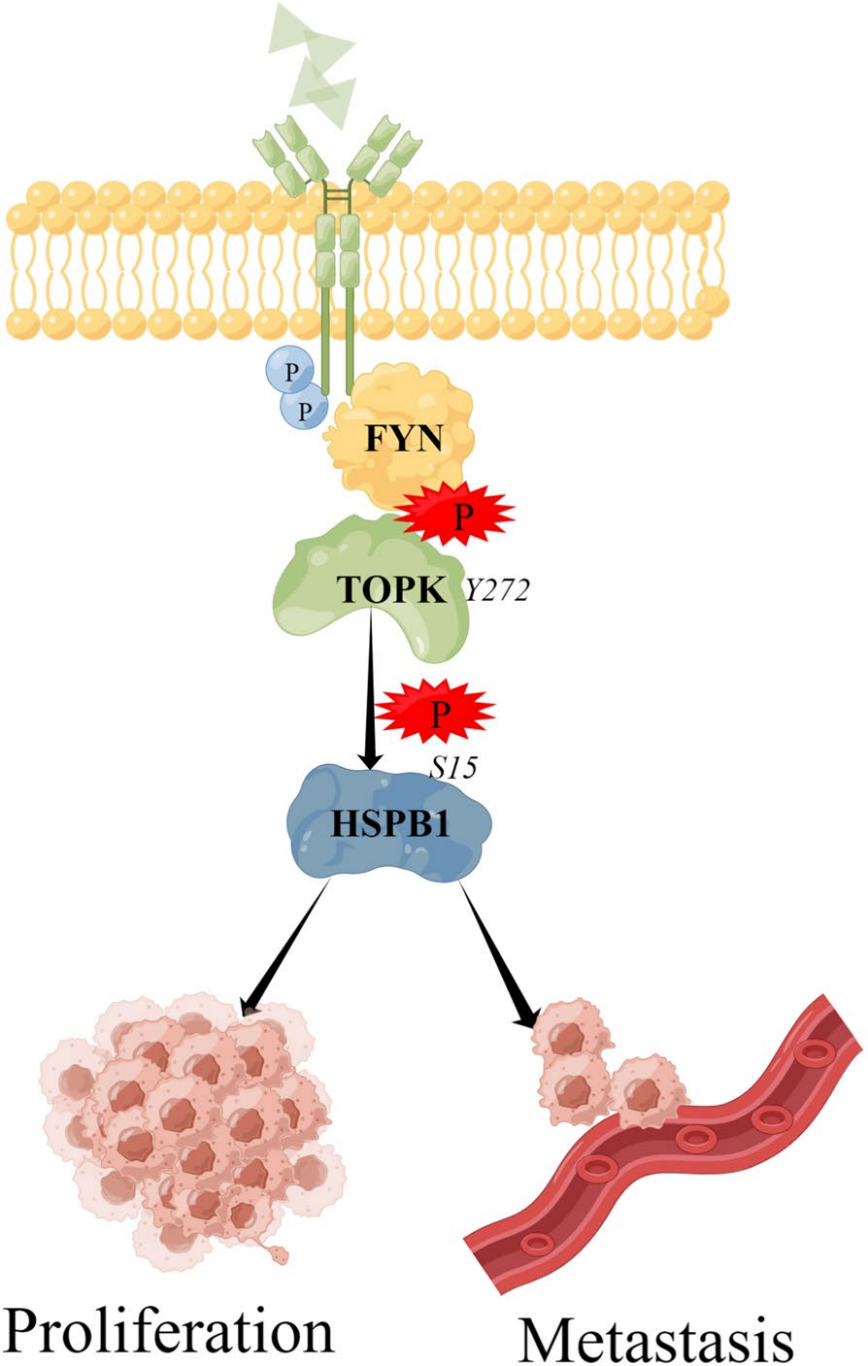
Diagram of molecular mechanism (By Figdraw)

In summary, we established FYN as a pivotal oncogenic factor in GC. This study not only elucidated the role of the FYN/TOPK/HSPB1 cascade in facilitating GC progression, but also identified a new site Y272 where TOPK can be phosphorylated by FYN (Fig. 9). These findings provide new targets for precision treatment of GC and have enormous value for clinical application.

## Supplementary Information

**Additional file 1: Supplementary Figure 1.** FYN expression is upregulated in TCGA GC. A-F. Differential expression of FYN in T-stage, N-stage, M-stage, pathological stage, age, and gender. G-H. The related pathways correlated with high FYN expression by GSEA (p < 0.05). I. FYN protein expression in the human protein altas database is higher in gastric cancer tissues than in normal tissues. **Supplementary Figure 2.** Differential protein motif analysis after silencing of TOPK. A. Flow chart of proteomics and phosphoproteomics analysis. B. Analysis of COG and KOG entries of differentially phosphorylated proteins. C. Differential phosphorylation protein GO terms analysis. D-E. S motif and T motif analysis summary. **Supplementary Figure 3.** Differential phosphorylated protein enrichment signaling pathway. A-C. Differential phosphorylation protein biological processes, molecular function and protein domain enrichment analysis. D-F. Differential phosphorylation protein signaling pathway enrichment analysis, the main enrichment signaling pathways were HIPPO signaling pathway, VEGF signaling pathway and SPLICEOSOME signaling pathway. **Supplementary Figure 4.** TOPK expression is upregulated in TCGA GC patients. A. The TOPK genetic alterations (gene amplification, deep deletion, or somatic mutation) and mRNA expression in GC samples from the TCGA cohort (total alteration rate: 14%). B-H. TOPK mRNA expression is upregulated in the tumor tissues compared with it in normal tissue group from TCGA and expression differences in different T-stage, N-stage, M-stage, pathological stage, age, and gender.

## Data Availability

The mass spectrometry proteomics data have been deposited to the ProteomeXchange Consortium via the PRIDE partner repository with the dataset identifier PXD036814. All other relevant data are already available as Supplementary material.

## Abbreviations

GC: Gastric Cancer
TOPK: T-lymphokine-activated killer-cell-originated protein kinase
HSPB1: Heat shock protein beta-1
COIP: Co-Immunoprecipitation
