# Supplementary material for: FYN/TOPK/HSPB1 axis facilitates the proliferation and metastasis of gastric cancer
Source: J Exp Clin Cancer Res. 2023 Apr 4;42:80. doi: 10.1186/s13046-023-02652-x (PMC10071617; doi:10.1186/s13046-023-02652-x)

**Supplemental Figure Legends**

**Supplementary Figure 1. FYN expression is upregulated in TCGA GC**

A-F. Differential expression of FYN in T-stage, N-stage, M-stage, pathological stage, age, and gender. G-H. The related pathways correlated with high FYN expression by GSEA (p < 0.05). I. FYN protein expression in the human protein altas database is higher in gastric cancer tissues than in normal tissues.


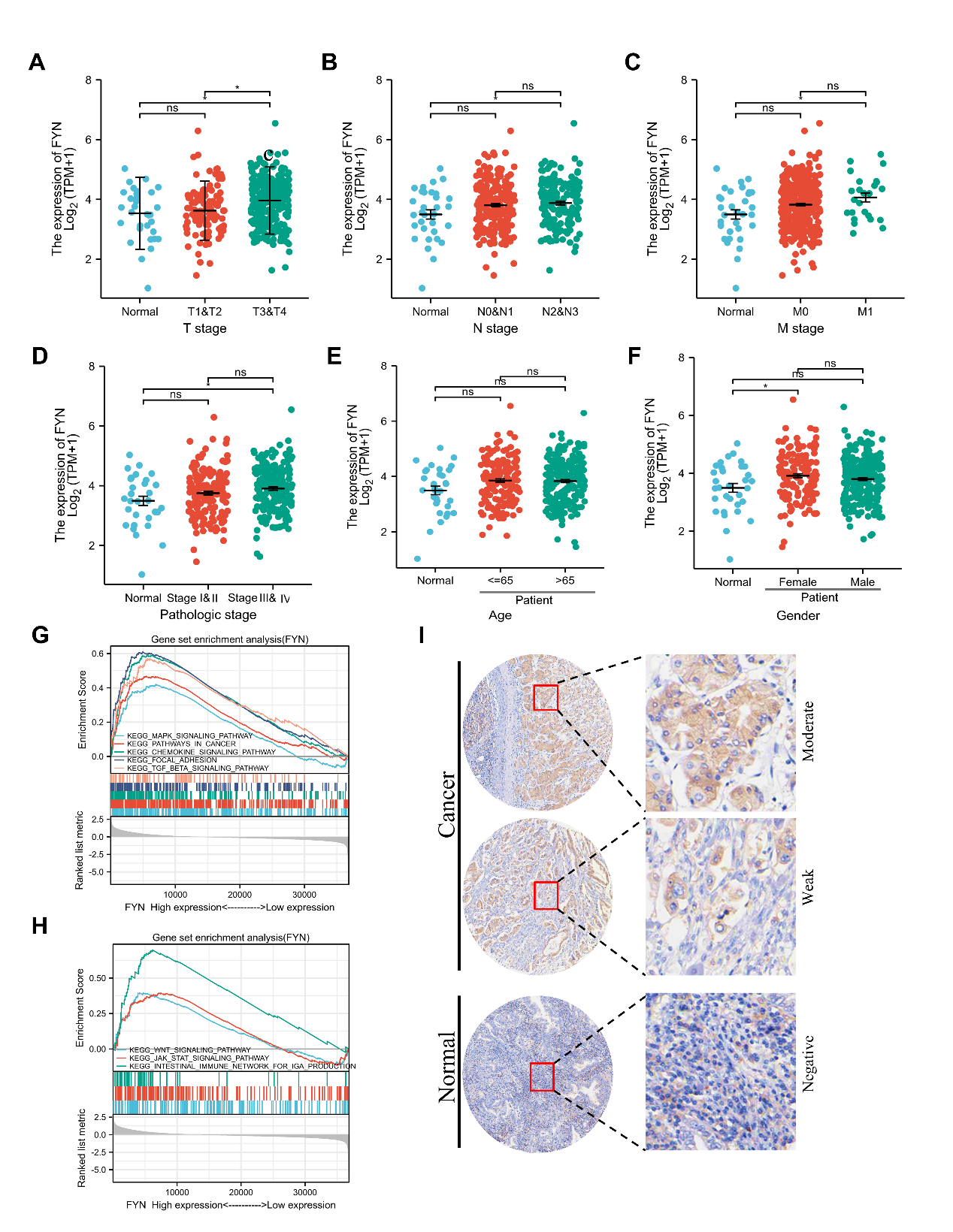


**Supplementary Figure 2.** **Differential protein motif analysis after silencing of TOPK.**

A. Flow chart of proteomics and phosphoproteomics analysis. B. Analysis of COG and KOG entries of differentially phosphorylated proteins. C. Differential phosphorylation protein GO terms analysis. D-E. S motif and T motif analysis summary.


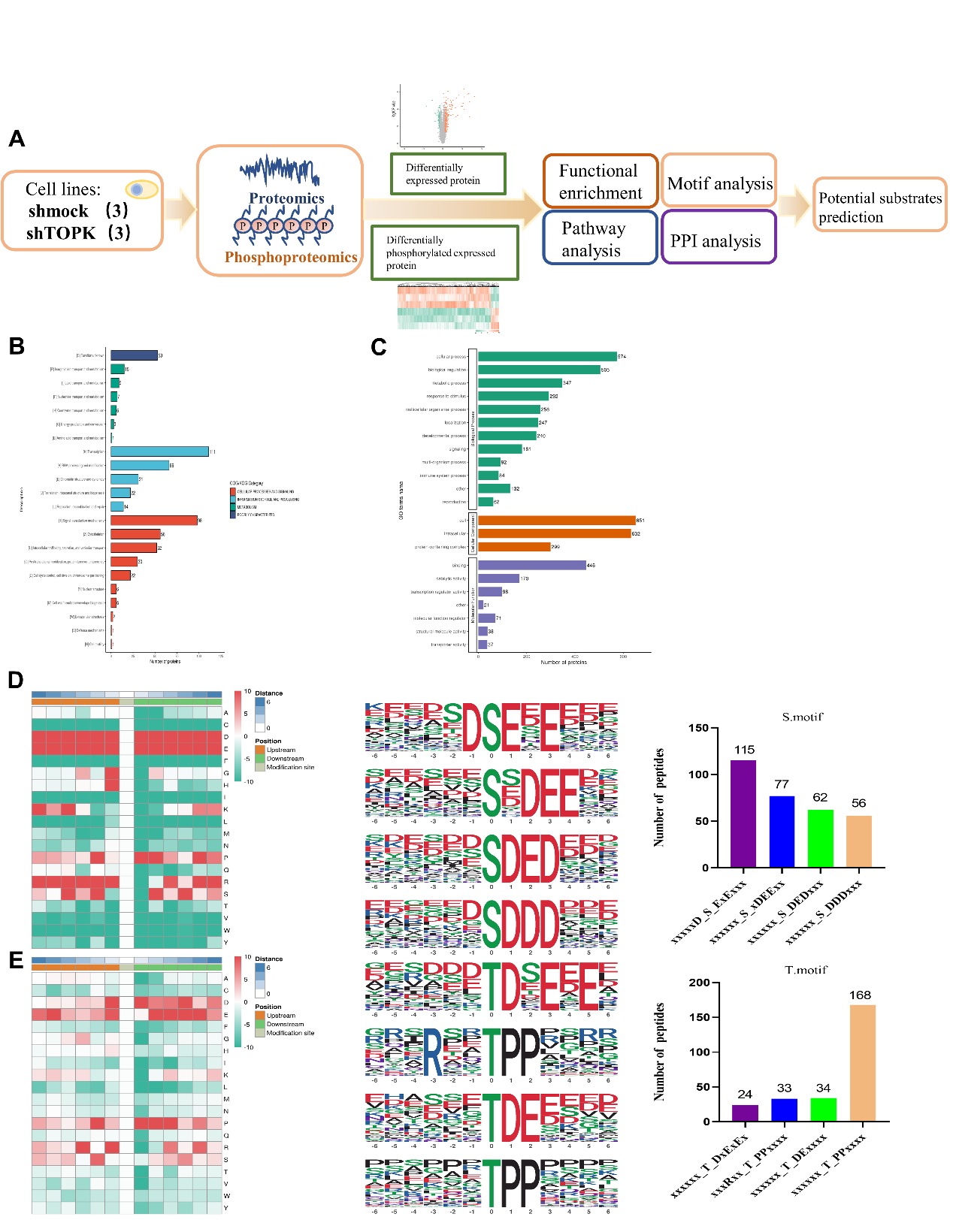


**Supplementary Figure 3.** **Differential phosphorylated protein enrichment signaling pathway.**

A-C. Differential phosphorylation protein biological processes, molecular function and protein domain enrichment analysis. D-F. Differential phosphorylation protein signaling pathway enrichment analysis, the main enrichment signaling pathways were HIPPO signaling pathway, VEGF signaling pathway and SPLICEOSOME signaling pathway.


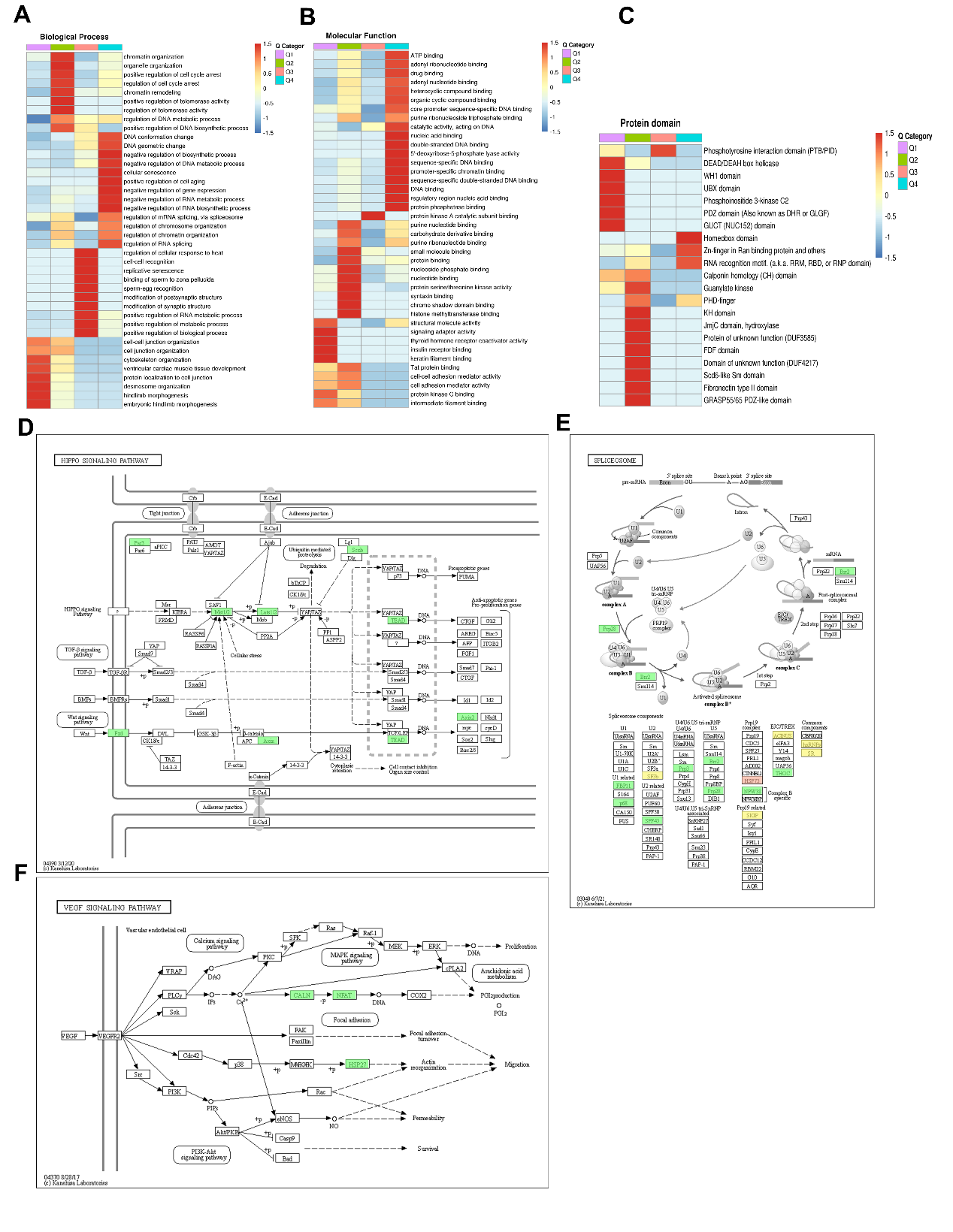


**Supplementary Figure 4.** **TOPK expression is upregulated in TCGA GC patients.**

A. The TOPK genetic alterations (gene amplification, deep deletion, or somatic mutation) and mRNA expression in GC samples from the TCGA cohort (total alteration rate: 14%). B-H. TOPK mRNA expression is upregulated in the tumor tissues compared with it in normal tissue group from TCGA and expression differences in different T-stage, N-stage, M-stage, pathological stage, age, and gender.


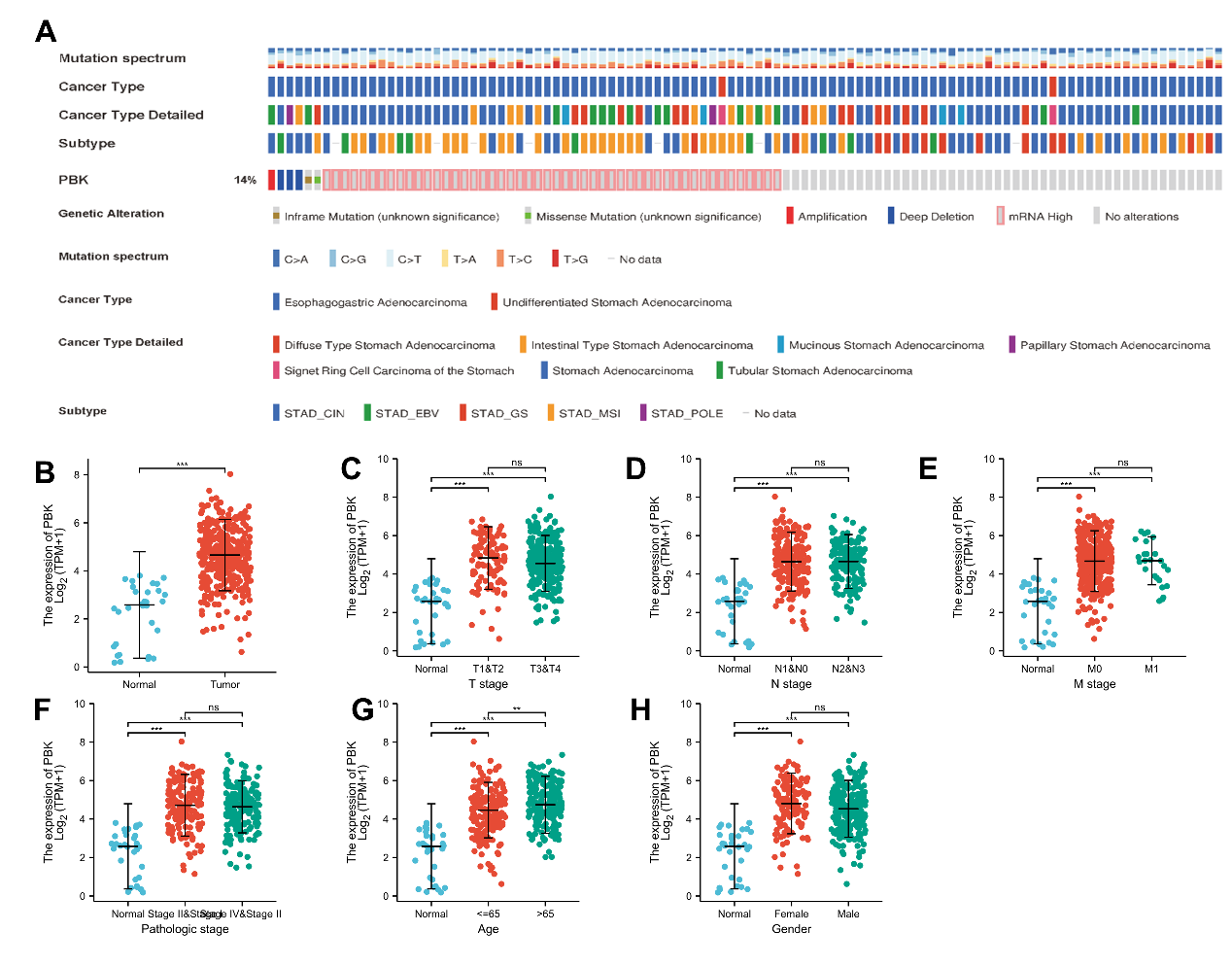

Supplement: Supplementary file 1 — Additional file 1: Supplementary Figure 1. FYN expression is upregulated in TCGA GC. A-F. Differential expression of FYN in T-stage, N-stage, M-stage, pathological stage, age, and gender. G-H. The related pathways correlated with high FYN expression by GSEA (p < 0.05). I. FYN protein expression in the human protein altas database is higher in gastric cancer tissues than in normal tissues. Supplementary Figure 2. Differential protein motif analysis after silencing of TOPK. A. Flow chart of proteomics and phosphoproteomics analysis. B. Analysis of COG and KOG entries of differentially phosphorylated proteins. C. Differential phosphorylation protein GO terms analysis. D-E. S motif and T motif analysis summary. Supplementary Figure 3. Differential phosphorylated protein enrichment signaling pathway. A-C. Differential phosphorylation protein biological processes, molecular function and protein domain enrichment analysis. D-F. Differential phosphorylation protein signaling pathway enrichment analysis, the main enrichment signaling pathways were HIPPO signaling pathway, VEGF signaling pathway and SPLICEOSOME signaling pathway. Supplementary Figure 4. TOPK expression is upregulated in TCGA GC patients. A. The TOPK genetic alterations (gene amplification, deep deletion, or somatic mutation) and mRNA expression in GC samples from the TCGA cohort (total alteration rate: 14%). B-H. TOPK mRNA expression is upregulated in the tumor tissues compared with it in normal tissue group from TCGA and expression differences in different T-stage, N-stage, M-stage, pathological stage, age, and gender. [file 13046_2023_2652_MOESM1_ESM.docx]
